# Bonds between body, face, and eyes reading

**DOI:** 10.1192/j.eurpsy.2021.1958

**Published:** 2021-08-13

**Authors:** K. Böck, V. Romagnano, J. Kubon, A. Sokolov, A. Fallgatter, M. Pavlova

**Affiliations:** 1 Department Of Psychiatry And Psychotherapy, Eberhard Karls University of Tübingen, Tübingen, Germany; 2 Psychiatry And Psychotherapy, Eberhard Karls Universitaet Tuebingen, Tuebingen, Germany

**Keywords:** Non verbal social cognition, Body language, Face language, Gender

## Abstract

**Introduction:**

Covering our faces with masks, due to COVID-19 pandemic safety regulations, we can no longer fully rely on the social signals we are used to. We have to read what’s between the lines. This is already difficult for healthy individuals, but may be particularly challenging for individuals with neuropsychiatric conditions.

**Objectives:**

Our main goal was to examine (i) whether capabilities in body and face language reading are connected to each other in healthy females and males; and (ii) whether capabilities to body/face language reading are related to other social abilities.

**Methods:**

Healthy females and males accomplished a task with point-light body motion portraying angry and neutral locomotion along with a task with point-light faces expressing happiness and angriness. They had to infer emotional content of displays. As a control condition, perceivers were administered with the RMET-M (Reading the Mind in the Eyes Test, Modified) with static images.

**Results:**

Females excelled on inferring emotions from body locomotion. Moreover, only in females, inferring emotions from body and face were firmly linked, whereas in males, face reading was connected to performance on the RMET-M.

**Conclusions:**

The outcome points to gender-specific modes in social cognition: females rely upon merely dynamic cues in facial and bodily displays, whereas males most likely trust configural information. The findings are of value for investigation of face/body language reading in neuropsychiatric conditions, most of which are gender specific.

**Disclosure:**

No significant relationships.

